# Assisted Suicide and Suicide Prevention: Ethical Perspectives, Attitudes and Challenges for Nurses in Long-Term Care—A Qualitative Focus Group Study

**DOI:** 10.3390/healthcare13243263

**Published:** 2025-12-12

**Authors:** Karen Klotz, Pia Madeleine Haug, Thomas Heidenreich, Eva-Maria Stratmann, Erik Jacob, Annette Riedel

**Affiliations:** Faculty of Social Work, Education and Nursing Sciences, Esslingen University of Applied Sciences, Flandernstraße 101, 73732 Esslingen, Germany; pia-madeleine.haug@hs-esslingen.de (P.M.H.); thomas.heidenreich@hs-esslingen.de (T.H.); eva-maria.stratmann@hs-esslingen.de (E.-M.S.); erik.jacob@hs-esslingen.de (E.J.); annette.riedel@hs-esslingen.de (A.R.)

**Keywords:** assisted suicide, voluntary assisted dying (VAD), medical assistance in dying (MAiD), desire to die, suicide prevention, ethics, ethical challenges, moral values, long-term care, nurses

## Abstract

**Background/Objectives**: Assisted suicide and suicide prevention remain subjects of intense societal, political, and professional-ethical debate in Germany. Nurses working in residential and home-based long-term care (LTC) play a pivotal role in responding to requests for assisted suicide and in supporting suicide prevention. While international research has explored diverse ethical perspectives and challenges related to these issues, little is known about how LTC nurses in Germany experience and navigate them. This study examines German LTC nurses’ ethical perspectives on assisted suicide and suicide prevention and explores the associated ethical challenges. **Methods**: A qualitative design employing both in-person and online focus groups was used. Data were analyzed following Mayring’s qualitative content analysis. **Results**: Twelve focus groups with a total of 96 nurses working in residential and home-based LTC were conducted between February and September 2025. Findings show that nurses perceive assisted suicide and suicide prevention as ethically complex and emotionally demanding. Three overarching themes emerged: (1) Intuitive and Emotional Reactions, (2) Ethical Perception and Ethical Reflection, and (3) Ethical Challenges. **Conclusions**: This study offers new insights into the diverse ethical perspectives of German LTC nurses on assisted suicide and suicide prevention. It extends existing knowledge through its explicit focus on the ethical issues and implications involved, both in residential and home-based LTC. The ethical challenges identified may enhance understanding of the factors underlying the development of moral distress in Germany and other countries where assisted suicide is a legal option. To help nurses navigate these ethically demanding situations, strategies at multiple levels are required. These include continuous ethics education, an open ethical culture, role definitions and clear professional guidance, alongside societal support for equitable access to general healthcare and suicide prevention services.

## 1. Introduction

Research has shown that end-of-life care poses significant ethical challenges for healthcare professionals, including nurses [1,2,3]. These challenges become particularly pronounced in the context of care recipients expressing a desire to die [1,2,4]. Such a desire is understood as a subjective, dynamic, multidimensional, and complex phenomenon, encompassing a spectrum of meanings—from the mere acceptance of one’s own death (without any intention of hastening death) to acute suicidality [4,5,6,7,8,9]. The underlying motives are equally diverse, ranging from physical, psychological and social to spiritual and existential dimensions [5,6,8,9]. Requests for assisted suicide and euthanasia may constitute *one* manifestation of a desire to die [5]. Both practices involve the intentional ending of a person’s life “at that person’s voluntary and competent request” [10] (p. 108), with the involvement of another individual, often a healthcare professional. The key distinction lies in who administers the life-ending medication: In assisted suicide, the individual self-administers the lethal dose, whereas in euthanasia, the medication is directly administered by the healthcare professional [10]. The term “Medical Assistance in Dying” (MAiD) will hereafter be used as an umbrella term encompassing both practices, unless a specific distinction is required.

Globally, an increasing number of countries are moving toward the legalization of MAiD [11], fueling ongoing debate given the ethical controversy surrounding the issue [11,12,13]. In Germany, assisted suicide (“Suizidassistenz”) is recognized as a legally permissible option since the landmark ruling of the Federal Constitutional Court on 26 February 2020, which emphasized the importance of the concept of “Freiverantwortlichkeit”. This concept requires that a person’s decision is based on a genuinely autonomous and well-considered choice, where the individual carefully weighs the pros and cons in light of their own values. The decision must be made free from acute mental illness, coercion, or manipulation, and it should demonstrate lasting inner firmness and stability [14]. To date, euthanasia (“Tötung auf Verlangen”) remains prohibited in Germany. Parallel to discussions on assisted suicide, suicide prevention has gained increasing political, societal and professional significance: In 2024, the Federal Ministry of Health published a national suicide prevention strategy [15] and the Federal Cabinet approved a draft law on suicide prevention [16]. The concept of suicide prevention is generally understood as a collective societal responsibility, encompassing universal measures targeting the general population, selective measures aimed at high-risk groups, and indicated measures directed at individuals at acute risk of suicide [17,18,19]. In light of the legal and political developments outlined above, the nursing and medical community in Germany continues to grapple with balancing the legal option of assisted suicide against the responsibility for suicide prevention—a tension that is particularly relevant for healthcare professionals [20,21,22,23,24,25].

Nurses, in particular, often serve as the first point of contact when care recipients express a desire to die [20,26,27,28]. This applies particularly to residential and home-based long-term care (LTC) of older adults [21,29,30,31,32], as suicide, suicidal ideation [33,34,35,36], and the desire to die are relatively common in this population [37,38]. Moreover, international [39,40,41] and German [42] data indicate that it is predominantly older adults in need of care who pursue MAiD. Within LTC, nurses carry particular responsibilities: They spend substantial time with care recipients and frequently build long-standing, caring and trust-based relationships [21,25,32]. When confronted with requests for MAiD, nurses are expected to respond professionally. This entails respecting and taking such requests seriously, while at the same time upholding their professional and ethical responsibility for suicide prevention [21,24,25,32]. In navigating these ethically complex and contentious situations, nurses are guided by professional values and core responsibilities of nursing, including “to alleviate suffering and promote a dignified death” [43] (p. 2). However, nurses’ experiences with MAiD are influenced not only by professional values and professional ethical standards, but also by personal convictions, organizational frameworks, institutional and societal attitudes [26,44,45]. A systematic review found that nurses’ support for MAiD is generally lower than that of the general public [11]. Consequently, international research shows that nurses encounter a wide range of ethical challenges related to MAiD, which can vary depending on the legal framework [20,44,46], and may also face ethical dilemmas in the context of suicide prevention [47,48]. Thus, nurses may require moral self-efficacy, understood as the confidence that one’s own decision-making and action competencies are sufficient to actively and successfully manage ethically challenging situations [49,50,51,52].

To date, little is known about the ethical perspectives, attitudes and challenges nurses face regarding assisted suicide and suicide prevention in LTC in Germany [20]. Existing research has primarily examined nurses’ experiences with assisted suicide and suicide prevention in palliative and hospice care [53,54,55] or their general experiences across various settings [56]. Only a few studies have focused specifically on LTC; those that exist primarily address encounters with desire to die in residential LTC within faith-based organizations [57] or residential LTC nurses’ general attitudes toward assisted suicide [58]. Against this background, it is particularly important to investigate the *ethical* dimensions of assisted suicide and suicide prevention in both *residential and home-based* LTC in Germany, taking into account the perspectives of nurses working across organizations of different types (i.e., faith-based vs. non-faith-based). This approach enables a more comprehensive understanding of the diverse ethical viewpoints and challenges, thereby contributing to the advancement of current knowledge.

The aim of this study is to address this gap by examining German nurses’ ethical perspectives and attitudes on assisted suicide and suicide prevention in residential and home-based LTC, and by identifying the related ethical challenges. It intends to answer the following research question: What are the ethical perspectives, attitudes and ethical challenges of nurses in residential and home-based LTC of older adults regarding assisted suicide and suicide prevention?

## 2. Materials and Methods

### 2.1. Study Design

The study was preregistered on the German Clinical Trials Register [59]. An explorative qualitative design was employed, using in-person and online single focus groups [60]. This approach was deemed particularly appropriate for exploring the ethical perspectives and related challenges, as it enables the capture of a wide range of participant opinions, perceptions, ideas, and feelings [61] and fosters discussion and debate on research topics that require insight into the meanings, experiences, and beliefs underlying participant views [60]. To ensure transparency in reporting, the Consolidated Criteria for Reporting Qualitative Studies (COREQ) [62] were applied (Supplementary File S1).

### 2.2. Ethical Considerations

Ethical approval for the focus groups was obtained from the Ethics Committee of the German Society of Nursing Science prior to their commencement (ethics proposal number: 24-035). Informed consent was obtained from each participant. Participation was entirely voluntary, and participants could withdraw at any time without providing a reason. All data were anonymized and stored securely for transcription purposes, accessible only to the research team.

### 2.3. Participants and Setting

To be eligible, participants had to have completed a minimum of a three-year nursing education program and to be currently employed in either residential or home-based LTC of older adults in one of six cooperating organizations. Furthermore, participants were required to have sufficient proficiency in German to actively engage in discussions and to provide informed written consent.

By LTC, we refer specifically to formal LTC, meaning care provided by trained professionals such as nurses. According to the World Health Organization, this “includes a package of services aimed at preventing, mitigating, and rehabilitating functional decline, delivered in a variety of settings” [63] (p. 5). In this study, we focus on residential and home-based formal LTC services, which are most commonly required by older adults [63].

As recommended for focus groups, we used a non-probability purposive sampling strategy [60,64]. Participants were recruited from both residential and home-based LTC of older adults and included both direct care providers and staff with broader responsibilities (e.g., quality management, leadership, practice education). Moreover, participants were drawn from six organizations of varying sizes and ownership structures (faith-based, non-faith-based, non-statutory welfare, municipal, and private organizations), in both urban and rural areas and across different federal states in Germany.

Recruitment was facilitated through designated contact persons in cooperating organizations who were informed that a diverse sample—in terms of age, professional experience, gender, religious beliefs, and cultural background—was desirable in order to capture a wide range of perspectives on the research topic.

Prior to the focus groups, participants received an information package containing:

1. An overview of the research project;
2. Details on the topic and objectives of the focus groups;
3. Formal information about session duration and structure;
4. A description of participants’ rights;
5. Contact details of the research team and the designated organizational contact person.

### 2.4. Data Collection

In-person and online single focus groups (no repeats) [60], were conducted in residential or home-based LTC between February and September 2025 (Table 1). Each group consisted of five to twelve nurses, allowing participants sufficient space to share their experiences while also enabling the exploration of diverse perspectives [61]. The in-person focus groups were conducted in quiet, confidential rooms provided by the cooperating organizations. For these, six participants withdrew prior to commencement: two due to illness, one for unknown reasons, one because they forgot the session, and two owing to short-notice shift coverage. To accommodate participants, two focus groups were held online via Webex, with nurses joining from a location of their choice, typically their home or workplace. In the online format, four participants withdrew—three for unknown reasons and one due to technical difficulties. Overall, focus groups ranged in duration from 110 to 190 min, with an average length of 153 min.

All focus groups were conducted using a pilot-tested interview guide developed by the research team, drawing on a comprehensive literature review and subject-matter expertise (Supplementary File S2). The interview guide aimed to address both nurses’ ethical and professional experiences with assisted suicide and suicide prevention—however, results on professional experiences will be reported elsewhere. Following the pilot phase, minor adjustments were introduced, and further minor adaptations were made during the sessions to respond to group dynamics while ensuring that the planned timeframe was respected. The guide comprised eight phases aimed at exploring nurses’ ethical and professional experiences with assisted suicide and suicide prevention in LTC. Each session began with a brief welcome and introduction (phase 1), followed by definitions of assisted suicide and suicide prevention to set the focus of discussion (phase 2). Participants were then asked to recall a situation in which they had received a request for assisted suicide—or, if not, a situation in which they encountered a desire to die (phase 3). Although the broader phenomenon of desire to die was not the main focus of the study, this step was designed to encourage participants to share personal experiences and to promote early engagement in the discussion [61]. Next, participants completed a short online survey on moral self-efficacy which guided self-reflection during the focus groups (phase 4, results will be reported elsewhere) and then noted their initial thoughts and feelings related to assisted suicide and suicide prevention (phase 5). The following phases invited discussion of professional and ethical challenges (phase 6), moral values and value conflicts (phase 7), and finally expectations and wishes for the future (phase 8).

Four female (K.K., P.M.H., A.R., E.M.S.) and one male (E.J.) author were involved in data collection and supported by three female research assistants. Each focus group was facilitated by 2–3 researchers, with a minimum of one moderating and one observing. Moderators guided the discussion using the interview guide, while observers took field notes on content and group dynamics [60]. Apart from the researchers and participants, no one else was present.

Due to the highly sensitive and controversial nature of the topic, no audio or video recordings were made. This decision was deliberate, aiming to protect participants’ privacy and confidentiality and to ensure that they could share their experiences openly without feeling inhibited or uncomfortable by unfamiliar recording devices. Researchers used a series of questions as outlined in the focus group interview guide (Supplementary File S2) to guide participants through the eight phases of the focus group. Participants first reflected on these questions individually, recording their personal experiences, thoughts, ideas, associations, resonances, and anticipations—either on facilitation cards, flip charts, and posters during in-person sessions or in the chat during online focus groups. This approach enabled participants to reflect on questions for discussion individually before contributing to the group discussion, thereby reducing the risk that their accounts would be influenced by other participants’ statements. Researchers photographed all facilitation materials from the in-person focus groups and saved the chat record from the online sessions. Before taking photographs/saving chat records, participants were invited at the end of each focus group to confirm that the materials accurately represented their experiences and to indicate whether they wanted to add or remove any content or comments. A researcher who was present during the entire focus group transferred participants’ verbatim written notes (i.e., their exact written words and sentences) from the facilitation material and chat records into a standardized transcript template immediately after each focus group to ensure consistent and accurate data recording. Whenever any written notes were difficult to read, a second researcher cross-checked them to guarantee precise and accurate transcription. The transcript template also included space for the field notes corresponding to each phase of the focus group. These field notes provided additional contextual information on the content of discussion and group dynamics intended to support the accurate interpretation of participant notes. To make efficient use of participants’ time, transcripts were not returned for comment, nor did participants provide feedback on the findings.

To determine whether the data collected were sufficient for answering the research question, the research team drew on the two distinct concepts of data saturation [60,61] and information power [65]. The principle of data saturation suggests that “focus group discussion sessions are run until a clear pattern emerges and subsequent groups produce no new information” [65] (p. 23). In this study, saturation was operationalized by continuously assessing whether newly collected data required the introduction of additional codes or whether the existing coding scheme remained stable. However, as saturation alone may be an insufficient rationale for determining the appropriate number of focus groups, the concept of information power as introduced by Malterud et al. [65] was applied. These authors suggest “that the size of a sample with sufficient information power depends on (a) the aim of the study, (b) sample specificity, (c) use of established theory, (d) quality of dialog, and (e) analysis strategy” [65] (p. 1753). In the present study, (a) the broad and exploratory research question, (b) the low level of sample specificity (as participants were included irrespective of prior experience with assisted suicide), (c) the exploratory design without reliance on an established theoretical framework, (d) the expected medium to high quality of dialog (supported by long focus group durations but moderated by researchers with moderate experience and reduced richness of collected data based on the data collection method), and (e) the analysis approach (qualitative content analysis requiring sufficient variation to form meaningful categories) indicate the need for a medium-to-larger number of focus groups covering both residential and home-based LTC. Based on the combined assessment of these distinct concepts, the research team concluded that after *n* = 12 focus groups with *n* = 96 participants, the data provided sufficient depth, variation and stability to adequately answer the research question.

### 2.5. Data Analysis

Data analysis was facilitated using MAXQDA (VERBI Software 2024) [66]. Mayring’s structuring content analysis was applied [67]. This strictly rule-based approach aims to systematically extract and summarize specific aspects from the data material. Following this method, categories, corresponding definitions, anchor examples, and coding rules were initially developed deductively based on the research question and theoretical considerations, and subsequently expanded inductively through analysis of the data [67]. Three of the authors (K.K., A.R., P.M.H.) immersed themselves in the data and pilot-coded three focus groups using the deductive coding system, adding inductive categories where necessary. The resulting codes were then discussed with a fourth researcher (T.H.). Based on these discussions and refinements, one researcher (K.K.) proceeded to code all focus groups using the updated coding scheme. All codes and findings were subsequently reviewed, discussed, and interpreted by four team members (K.K., P.M.H., A.R., T.H.). Additionally, the coding scheme and interpretations were presented to two independent researchers (E.M.S, E.J.), who had not been involved in the focus groups at this stage, to assess the appropriateness of both coding and analytical conclusions. Any disagreements on coding were resolved through discussion until consensus between all researchers (K.K., P.M.H., A.R., T.H., E.M.S., E.J.) was reached. An overview of the final coding scheme, detailing themes, categories, definitions, anchor examples and coding rules is provided in Table 2.

### 2.6. Research Team and Reflexivity

The research team comprised two project leaders—one professor of nursing science and ethics (A.R.) and one professor of psychology with a focus on nursing and social work (T.H.)—both experienced in qualitative research. Four additional researchers (K.K., P.M.H., E.M.S., E.J.) were employed as research associates at the time of the study, held a master’s degree in nursing and brought experience in group moderation as well as initial training in qualitative research methods acquired during their studies. Acknowledging the crucial role of moderator skills in the success of focus groups, the less experienced team members received targeted briefings from the project leaders, consistent with recommendations [60]. With the exception of T.H., who holds professional experience as a clinical psychologist, all researchers had professional backgrounds in nursing across various care settings. At the beginning of each focus group, participants were informed about the researchers’ professional backgrounds, the overarching project, and the researchers’ specific areas of interest. No prior relationships existed between the research team and the participants.

### 2.7. Trustworthiness

Several strategies were applied to strengthen the trustworthiness of the study, addressing key aspects such as credibility, transferability, dependability, and confirmability [68].

Credibility was strengthened through ongoing researcher reflexivity and prolonged engagement with participants during focus groups, which helped establish a good rapport [68]. Reflexivity was considered particularly important due to the sensitive and potentially controversial nature of the research topic. To support this, postscripts were written immediately after each focus group by all researchers present, allowing for the ongoing acknowledgment of researchers’ perspectives and potential biases. These postscripts documented information on the researchers (e.g., professional backgrounds, research experience) as well as reflections on: (1) group interactions, (2) moderation, (3) key content points, (4) researchers’ own feelings, values, and attitudes, (5) other observations, and (6) remarks related to the original objectives of the focus group and the methodology.

We aimed to increase transferability by providing a detailed description of our research context (i.e., description of German legal framework, participants/sampling procedure, setting/cooperating organizations, data collection and research team). This allows readers to judge the applicability of our findings to other settings and contexts [68].

To improve dependability, we kept an audit trail supplemented by research documents (e.g., different versions of participant handouts, postscripts, logbook on MAXQDA) to document methodological decisions, modifications and adaptations made throughout the research process [68].

Although member checking was not conducted in order to respect participants’ time, confirmability was ensured through systematic reflective journaling (postscripts), and complemented by continuous peer debriefing and team discussions [67].

**Table 2:** Coding Scheme, detailing the Coding Tree.

**Theme 1. Intuitive and Emotional Reactions** This theme encompasses participants’ initial moral intuitions and moral emotions related to assisted suicide and suicide prevention in care recipients who request assisted suicide.			
**Category**	**Definition**	**Anchor Example**	**Coding Rule**
**Moral Intuition**	Moral intuitions refer to immediate moral judgments, or instinctive reactions that arise spontaneously in a given situation, before conscious moral reasoning occurs [69,70,71].	“*Under no circumstances!*” (F6)	Code text segments in this category when participants express spontaneous emotions, or thoughts that reveal an immediate moral evaluation of a situation in the context of assisted suicide or suicide prevention.
**Moral Emotions**	Moral emotions are emotions that serve as a motivational force, providing the energy and drive necessary to act ethically and to refrain from wrongdoing [69,72].	“*I feel sad about challenges in the healthcare system in getting timely help during mental health crises*” (F3)	Code text segments in this category when the expressed emotion (e.g., guilt, shame) is described as motivating or discouraging ethical behavior, or when participants indicate that the emotion arose in response to a moral or ethical issue. General emotional expressions without moral relevance are not included.
**Theme 2. Ethical Perception and Ethical Reflection** This theme illustrates how participants demonstrated ethical sensitivity, identified the moral values at stake in relation to assisted suicide and suicide prevention in care recipients who request assisted suicide, and engaged in ethical reasoning by reflecting on their own moral stances toward these issues.			
**Category**	**Definition**	**Anchor Example**	**Coding Rule**
**Ethical Sensitivity**	Ethical sensitivity refers to the ability to recognize the moral significance of a situation. It is a source of ethical reflection and ethical decision-making [71,73].	“*Morality—how do I feel afterwards?*” (F7)	Code text segments in this category when participants show awareness of the moral relevance or ethical implications of a situation.
**Guiding Moral Values**	“Values in nursing are those ends sought by both the profession and in nurse—patient relationships. These include, for example, health, dignity, respect, compassion, equity, inclusivity.” [43] (p. 27)	“*respect*” (F1) “*self-determination*” (F9)	Code text segments in this category when participants refer to values that guide nursing practice or shape nurse—care recipient relationships and related care situations.
**Ethical Reasoning**	Ethical reasoning is the process by which individuals make decisions about what is right and wrong, based on ethical principles, values, social norms or theories that inform their arguments [74].	“*We deal with older people every day. For example, we have a 105-year-old here. She says she didn’t want to live that long; that wasn’t what she wished for. At some point, they are ready to go, and you have to accept that.*” (F11)	Code text segments in this category when participants explain or justify decisions, actions, or judgments by referring to ethical principles, values, social norms, or moral theories.
**Theme 3. Ethical Challenges** This theme captures the range of ethical challenges participants discussed in relation to assisted suicide and suicide prevention in care recipients who request assisted suicide, encompassing the moral controversy with its diverse ethical perspectives and value-based conflicts between stakeholders, as well as participants’ experiences of internal moral uncertainty and perceived moral constraints that at times impeded what they regarded as the ethically right course of action.			
**Category**	**Definition**	**Anchor Example**	**Coding Rule**
**Moral Controversy** This subtheme highlights the moral complexity and contentious nature of assisted suicide and suicide prevention in care recipients who request assisted suicide, arising from the sub-subthemes varying ethical perspectives held by different individuals and value-based conflicts between various stakeholders.			
**Varying Ethical Perspectives**	Varying ethical perspectives capture “the variation and complexity of […] perspectives” [75] (p. 2) of various stakeholders (e.g., nurses, care recipients, relatives, other healthcare professionals) on assisted suicide and, in our understanding, also on suicide prevention.	“*Recognition of the resident’s desire to die versus my own position as a nurse, not wanting to take an active or facilitating role in the dying process*” (F6)	Code text segments in this category when participants describe, compare, or reflect on different moral or ethical viewpoints of various stakeholders regarding a situation, decision, or practice (e.g., assisted suicide, suicide prevention), highlighting the variation or complexity of ethical reasoning.
**Value-Based Conflicts**	Value-based conflicts arise when two or more equally important moral values held by various stakeholders involved come into conflict and cannot be fulfilled simultaneously [76].	“*Confidentiality (resident)* vs. *truth (nurse)*” (F10)	Code text segments in this category when participants describe situations in which two or more moral values between various stakeholders are in conflict, making it impossible to fulfill all of them simultaneously.
**Internal Moral Uncertainty**	Internal moral uncertainty occurs, when a moral agent feels uncertain about what they themselves consider the ethically right course of action to take in a situation [77,78].	“*Shortening a person’s life—is it killing? Or helping? How do I deal with it myself?!!!*” (F6)	Code text segments in this category when participants express internal uncertainty or personal doubt about what they themselves consider the ethically correct course of action in a given situation (e.g., statements indicating hesitation, doubt, or indecision about what is ethically right).
**Moral Constraints**	Moral constraints arise when a moral agent is prevented/constrained from acting in the way they believe to be the most ethically justified [77,78].	“*One reaches one’s limits because, from a medical perspective, there is often little real support available—neurologists are overbooked and provide only medication-based treatment, whereas psychotherapy and similar interventions would in fact be more helpful.*” (F9)	Code text segments in this category when participants describe situations in which they are prevented or restricted from taking the action they believe to be ethically justified (e.g., situations where external factors block ethically preferred actions or expressions of being hindered from doing what is perceived as ethically right).

## 3. Results

### 3.1. Description of Study Sample

A total of *n* = 12 focus groups were conducted. *n* = 8 focus groups were conducted with nurses working in residential LTC facilities and *n* = 4 focus groups were conducted with nurses working in home-based LTC in the South (Baden-Württemberg) and Mid-West (North Rhine-Westphalia) of Germany (Table 1). *n* = 96 nurses participated in the focus groups, with *n* = 73 employed in residential LTC and *n* = 23 employed in home-based LTC. In light of the ethically sensitive nature of the research topic and to safeguard participants’ anonymity, no formal sociodemographic data were collected. Nonetheless, at the beginning of each focus group, participants were invited to provide a brief self-introduction, including information on their professional background, current role, and work experience. This contextual information provided the researchers with insight into the sample’s composition. Based on these introductions, the sample included both male and female nurses from diverse cultural backgrounds, with varying levels of professional experience in general nursing as well as LTC for older adults. Participants spanned a wide age range and included both direct care providers and staff with broader responsibilities (e.g., quality management, leadership, practice education).

### 3.2. Overview of Themes

This article reports on the findings concerning nurses’ ethical experiences—their ethical perspectives, attitudes and associated challenges in relation to assisted suicide and suicide prevention. Our analysis identified three major themes: (1) Intuitive and Emotional Reactions, (2) Ethical Perception and Ethical Reflection, and (3) Ethical Challenges (Table 2). These themes are presented using quotes drawn from participants’ written notes (e.g., from facilitation materials or chat records), complemented by our interpretive synthesis and contextualization based on the verbal discussions captured in our field notes.

### 3.3. Theme 1. Intuitive and Emotional Reactions

#### 3.3.1. Moral Intuition

When reflecting on their initial reactions to requests for assisted suicide, nurses reported a range of intuitive responses. Some reactions reflected strong refusal, as indicated in written statements such as “*Under no circumstances!*” (F6) or “*I’m not willing to do that*” (F6). Other written notes included single-word expressions like “*disapproval*” (F6), feeling “*powerless*” (F12), “*uneasy*” (F12), “*shocked*” (F5), “*cautious*” (F4) and “*aghast*” (F6). Additional responses included stress and uneasiness, as reflected in notes such as “*I feel stressed*” (F3) and “*the expected uneasiness*” (F10). Nurses later explained verbally that these statements reflected their immediate, spontaneous and initially unreflective reactions to the topic of assisted suicide.

When considering initial thoughts and feelings on suicide prevention, nurses recorded qualitatively similar reactions of feeling “*powerless*” (F4), “*astonished*” (F8), “*irritated*” (F8), “*nervous*” (F2) and “*cautious*” (F6). However, these responses were written and discussed less frequently than those related to assisted suicide.

#### 3.3.2. Moral Emotions

In response to requests for assisted suicide, nurses reported a range of moral emotions. Some participants recorded feeling “*anxious*” (F7), explaining verbally that assisted suicide conflicts with their religious or cultural beliefs that life should end naturally. Others noted feeling worried, writing, “*When I think about requests for assisted suicide, I feel worried because the person might act on their wishes*” (F7). Feelings of “*helplessness*” (F7), “*powerlessness*” (F7) and “*sadness*” (F4) were common, particularly when nurses felt unable to relieve suffering. One participant wrote, “*A request for assisted suicide would make me very sad, because I would ask myself what had happened. What reason does the person have for no longer wanting to live?*” (F11). Others recorded feeling “*unhappy*” (F4), explaining that this emotion arose when they felt unable to intervene. Some even noted feeling “*guilty*” (F6), adding during the discussion that this guilt emerged when a care recipient under their responsibility requested assisted suicide.

When reflecting on suicide prevention, similar emotions emerged. Nurses wrote down that they experience a “*feeling of powerlessness*” (F8) or feeling “*helpless because one often becomes aware of the problems too late*” (F3), highlighting in discussion that these situations evoke strong emotional distress and a sense of moral responsibility that can be difficult to manage. Participants also wrote down that they felt “*sad*” (F6), “*down*” (F12) or “*angry*” (F6) when insufficient time was available for care recipients and emphasized systemic barriers, for example, “*I feel sad about challenges in the healthcare system in getting timely help during mental health crises*” (F3).

### 3.4. Theme 2. Ethical Perception and Ethical Reflection

#### 3.4.1. Ethical Sensitivity

Nurses described requests for assisted suicide in general terms as an ethical topic, which highlights their ethical sensitivity. They wrote down thoughts and questions such as “*Ethics?*” (F2) or “*Morality—how do I feel afterwards?*” (F7) and verbally elaborated on the moral dimensions inherent in these requests. This reflection prompted them to assess their own emotional readiness, as indicated in written statements like, “*Do I feel emotionally stable enough to fulfill this person’s final wish?*” (F9), with verbal emphasis on the importance of protecting their own well-being. At the same time, nurses acknowledged their moral responsibility, noting comments such as, “*I am responsible for whether the resident lives or dies*” (F7), and having to “*Ask about reasons for the desire to die*” (F4). Participants also discussed the moral importance of dialog and involving relatives, reflected in written notes like “*Was this discussed with the family?*” (F2).

In the context of suicide prevention, nurses emphasized the importance of safeguarding patient rights. For example, one participant wrote, “*I keep in mind that the person’s wish takes priority*” (F12), while another stressed the importance of preserving self-determination: “*preserve the resident’s/patient’s self-determination*” (F9). They recognized the moral weight of their actions, by discussing written notes such as “*incorrect actions can lead to a deterioration of the resident’s situation*” (F9) or “*I can intervene to help, but I could also make things worse*” (F7). Nurses reflected on the personal impact of these situations. They emphasized the importance of conveying the care recipient’s value, by writing statements such as “*You are important to me*” (F6). They further discussed the benefits of collaborative approaches, highlighting comments like “*Seek collaborative solutions, including family*” (F4) and emphasized the value of structured discussions.

#### 3.4.2. Guiding Moral Values

Nurses described a wide range of moral values they regarded as important in relation to assisted suicide and suicide prevention in care recipients who request assisted suicide, often adopting the perspectives of various actors involved.

For nurses themselves values that were frequently mentioned in writing or discussion include “*respect*” (F1; F11), “*empathy*” (F2; F4), “*responsibility*” (F8; F5), “*dignity*” (F10; F4), “*collaboration*” (F7; F4) and “*compassion*” (F6; F5), while “*care*” (F9; F12), “*safety*” (F3; F4), “*trust*” (F1; F4), and “*confidentiality*” (F10; F5) were discussed in some focus groups, and “*acceptance*” (F6) was mentioned by a few nurses in one focus group.

Regarding care recipients, nurses considered “*self-determination*” (F9; F4) and “*dignity*” (F9; F11) commonly as the most crucial moral values in their written notes and discussions. Additional, though somewhat less prominent, values mentioned included “*respect*” (F2; F5), “*freedom*” (F6; F11), “*privacy*” (F10; F4), “*quality of life*” (F7; F12), “*confidentiality*” (F3; F10), “*trust*” (F8; F4), or the principle of “*autonomy*” (F2; F11).

For relatives, the values deemed most important in participant notes and discussion were “*compassion*” (F2; F11), “*care*” (F3; F12), “*respect*” (F1; F4) and “*trust*” (F6; F5), whereas “*empathy*” (F9; F5), “*responsibility*” (F3; F4), “*collaboration*” (F7), “*truth*” (F10; F5), “*dignity*” (F6; F4), “*fairness*” (F8; F10) and “*safety*” (F8; F4) were mentioned less frequently.

Finally, for other professional groups involved (e.g., physicians or those not further specified), “*collaboration*” (F7; F5) was noted and discussed commonly, as well as “*responsibility*” (F3; F8), “*respect*” (F8; F5), “*dignity*” (F3; F4) and “*trust*” (F3; F8).

#### 3.4.3. Ethical Reasoning

Nurses demonstrated that they engage in ethical reasoning when reflecting on their own attitudes towards assisted suicide. A variety of viewpoints emerged. For some, assisted suicide was morally unacceptable because it was considered equivalent to killing, as reflected in written notes such as: “*I don’t want to end a life*” (F6). Through the discussions, it became evident that for other participants, the acceptability of assisted suicide was closely tied to the care recipient’s age, was highly situation-dependent, and influenced by whether they could understand the wish. This is reflected in one participant’s written comment: “*It depends on the situation and the person. For example, someone who is seriously ill, alone, and 85 years old. I wouldn’t try to convince a person like that to keep living. […]. So, I wouldn’t encourage it or insist on it, but I would understand*” (F11). Nurses who ethically supported assisted suicide often based their reasoning on the desire to reduce suffering, ensure a peaceful death, or to accept a care recipients wish without further questioning.

In the context of suicide prevention, nurses also engaged in ethical reasoning, although much less frequently compared to assisted suicide. Some verbally explained that they viewed suicide prevention as their primary ethical responsibility, and referred to written notes such as “*The topic of suicide prevention is at the forefront for me*” (F2) or “*I feel responsible*” (F5) to underscore their reasoning. Others emphasized that the ethical appropriateness of suicide prevention was highly situation-dependent. Still others questioned whether suicide prevention was always ethically justified, as one participant wrote: “*What if the resident’s wishes differ? There are limits*” (F7).

### 3.5. Theme 3. Ethical Challenges

#### 3.5.1. Moral Controversy

##### Varying Ethical Perspectives

One ethical challenge that emerged was the complex task of navigating the diverse ethical perspectives of various stakeholders involved in assisted suicide. From nurses’ perspectives, this challenge was shaped by various relational dynamics. First, nurses noted the tension between care recipients’ wishes for assisted suicide and socially accepted moral norms. One group collectively summarized this on a facilitation card as: “*Self-determination* vs. *societal value norms, which may not align with individual beliefs*” (F5). Several nurses reflected on the personal dilemma of reconciling their own moral convictions with their professional responsibilities towards care recipients who hold different moral views, as recorded in written notes such as: “*Recognition of the resident’s desire to die versus my own position as a nurse, not wanting to take an active or facilitating role in the dying process*” (F6). At times, these differing perspectives also strained the nurse-care recipient relationship. One participant wrote: “*She reacted with offense when the staff explained they could not assist her and expressed doubt about this as well*” (F6). Additionally, nurses pointed to the challenges of navigating conflicts that arose when different family members morally disagreed with each other, illustrated by written notes such as: “*Dealing with relatives when they disagree*” (F7). Intra- and interprofessional collaboration—particularly between nurses and physicians—was also affected by differing ethical positions.

These varying ethical perspectives were far less pronounced in discussions about suicide prevention. Some nurses explained that challenges arose when they did not personally regard suicide prevention as ethically appropriate but felt it was expected of them. Other challenges emerged in situations where nurses and relatives held conflicting views on the moral appropriateness of suicide prevention measures, summarized in a written note as: “*Conflict between the nurse and family members*” (F2). Finally, nurses discussed how differing perspectives on suicide prevention occasionally strained team dynamics, as one participant reflected: “*Colleagues showing a lack of seriousness and unwillingness to engage with the topic*” (F3). This participant further explained verbally that such behavior was experienced as ethically inappropriate.

##### Value-Based Conflicts

The participants reported a broad range of value-based conflicts in the context of requests for assisted suicide and suicide prevention in care recipients who request assisted suicide, which could involve any of the persons engaged in the situation.

When asked to identify one conflict that was particularly significant for the entire group, the most frequently discussed was the tension between values central to the care recipient and those central to the nurse. Recorded written examples include “*self-determination* vs. *care* vs. *quality of life*” (F4), “*confidentiality (resident)* vs. *truth (nurse)*” (F10) and “*dignity* vs. *self-determination*” (F2). Among these, however, “*care* vs. *self-determination*” (F3) was mentioned most frequently. Some participants further elaborated on this conflict, noting that the self-determination of care recipients may not only clash with nurses’ understanding of care, but also with that of relatives, specifying in their written comments: “*self-determination of residents* vs. *care of nurses* vs. *care of relatives*” (F8). On a broader level, participants emphasized that self-determination can also come into conflict with societal or religious value systems and summarized this in written notes such as: “*self-determination* vs. *societal value systems (which may differ from individual values)*”.

#### 3.5.2. Internal Moral Uncertainty

When discussing the practice of assisted suicide, it became apparent that nurses often experienced internal moral uncertainty about what they themselves considered as the ethically right course of action. One nurse reflected in writing: “*Shortening a person’s life—is it killing? Or helping? How do I deal with it myself?!!!*” (F6), highlighting their own internal uncertainty. Some discussed how they felt uncertain about context-specific ethics, referring to written questions such as: “*When is assisted suicide acceptable, and when is it ‘reprehensible’?*” (F8). For others, internal moral uncertainty centered on their own professional role and their rights to object. In certain cases, doubts were raised regarding the individual circumstances of a request, as illustrated by the written question, “*Has the affected person truly exhausted all medical options?*” (F1). Overall, nurses discussed feeling ill-prepared to navigate assisted suicide requests from an ethical perspective and to develop their own personal moral positions. They also voiced a need for additional support in the form of further education, training, and clear guidelines to reduce internal moral uncertainty.

Participants also described experiencing internal moral uncertainty in the context of suicide prevention, although this was reported less frequently. One nurse wrote, “*I feel uncertain*” (F8), while another noted, “*Who can I involve for support?*” (F5). Nurses discussed that they were not always sure which changes to the care plan were ethically appropriate. This uncertainty could be heightened when they wished to respect the care recipient’s wishes but found advance directives to be unclear. Some nurses expressed their internal moral uncertainty through the need for guidance, recording comments such as “*need for clear guidelines*” (F8) and verbally explaining that they hoped such guidelines could clarify the ethically right course of action. Others reflected on the uniqueness of each case, noting in written statements, “*There is no one-size-fits-all approach*” (F8) and explained that their internal moral uncertainty arose from the uniqueness of each care recipient’s case.

#### 3.5.3. Moral Constraints

When discussing assisted suicide, nurses reported facing several ethical challenges linked to external constraints. One nurse felt restricted by what she perceived to be the legal context, writing, “*Assistance to die is illegal in Germany*” (F4), although this perception does not fully reflect the current legal status. Others described that they would feel pressured if they were required to participate in assisted suicide despite their personal reluctance. Time constraints were another key limitation discussed, preventing nurses from engaging in dialog to the extend they felt was ethically required, summarized through written comments such as: “*There is often insufficient time for these discussions*” (F1).

Insufficient time was also perceived as a moral constraint in the context of suicide prevention. Many nurses referred to time limitations in their written comments. For example, one nurse emphasized the need for “*more time*” (F6), while another noted the limitations of “*time as a framework*” (F5), explaining how they felt they lack time as a resource to provide adequate preventive and quality nursing care. Beyond time constraints, nurses pointed to broader societal constraints, through discussing how the structure of the healthcare system can hinder suicide prevention. One participant summarized this in a written comment: “*One reaches one’s limits because, from a medical perspective, there is often little real support available—neurologists are overbooked and provide only medication-based treatment, whereas psychotherapy and similar interventions would in fact be more helpful*” (F9).

## 4. Discussion

### 4.1. Ethically Complex and Emotionally Demanding

For the following discussion, it is important to recognize that nursing qualifications and the legal frameworks governing MAID vary considerably across countries and settings [79]. As a result, findings and experiences from other contexts should be interpreted with caution. Nonetheless, there appears to be international consensus on key issues, including the right to conscientious objection and the provision of dignified care for all individuals, regardless of how they choose to die. Against this backdrop, our study findings are carefully interpreted and contextualized.

Our findings suggest that requests for assisted suicide constitute an ethically complex issue for nurses working in German residential and home-based LTC across various organizations, particularly given their simultaneous responsibility for suicide prevention, which has been at the center of political, professional and societal attention in recent years. Based on Monteverde’s definition [76], we understand ethical complexity as present in situations that are morally uncertain, carry a high risk of causing harm, involve interdependencies, or encompass multiple interconnected actors or interfaces.

It is noteworthy that nurses in our study exhibited both moral, intuitive, and emotional responses regarding assisted suicide and suicide prevention (Theme 1. Intuitive and Emotional Reactions). While international research on MAiD has documented intuitive and predominantly negative emotional responses, such as guilt or anger [20,29,80], some studies also report neutral or positive emotions, such as feeling honored, fulfilled or relieved, since suffering can be reduced [20,80,81]. In contrast, our analysis revealed only negative emotions, which may be attributed to our specific focus on *moral* emotions as defined in the coding scheme (Table 2), whereas other studies may have considered emotions in a broader sense. Moreover, nurses’ psychological well-being in relation to MAiD may be influenced by factors beyond solely moral reasons, such as legal uncertainty, fears of professional repercussions or stigma [81], their roles and level of involvement in MAiD, and the availability of support systems [27]. Furthermore, our findings indicate that intuitive and emotional responses were more pronounced for assisted suicide than for suicide prevention, although international studies also highlight nurses’ emotional labor in suicide prevention [47]. This suggests that emotional responses to suicide prevention may carry a different significance and may be less rooted in moral considerations than those elicited by assisted suicide requests. Considering that moral perspectives are shaped by cultural and societal contexts, and that societal values influence attitudes toward assisted suicide [13], one possible explanation for this pattern in our results is social desirability [82] because of the prominence of suicide prevention in contemporary political, societal, and professional debates in Germany, where it is more widely accepted and less controversial than assisted suicide [22,23,24].

Nurses in our study demonstrated ethical sensitivity, identified relevant moral values, and engaged in nuanced ethical reasoning regarding both assisted suicide and suicide prevention (Theme 2. Ethical Perception and Ethical Reflection). This aligns with international research on MAiD, which shows that nurses are acutely aware of the ethical complexity of MAiD requests [2,20,46], and though less extensively documented, also acknowledges their recognition of the ethical dimensions involved in suicide prevention [47,48]. Nurses in our study reflected deeply on the moral values at stake for all parties involved in both assisted suicide and suicide prevention. Consistent with prior research [83,84], respect for autonomy and self-determination was frequently identified as a central guiding value for nurses. Our findings further suggest that nurses’ ethical reasoning regarding assisted suicide is highly situation-dependent and closely tied to efforts to understand care recipients’ individual wishes, a pattern also reported in previous studies [20,26,27,56]. Notably, the age of care recipients seemed to shape nurses’ moral reasoning, with some showing greater acceptance of assisted suicide for older individuals. This finding raises an important ethical issue, as this line of reasoning may reflect implicit ageism, which could undermine the quality of care and holistic approaches to suicide prevention, potentially contributing to higher suicide rates among older adults in need of care [85,86]. Although the systematic review by Fernández-Puerta et al. [87] concluded that ageism among healthcare professionals tends to be low, the authors emphasize that attitudes and/or discriminatory structures may vary depending on the cultural context. Thus, to avoid normalization of MAiD in older adults, attention must be directed to age-related stereotypes, societal and professional attitudes and perceptions of aging, as well as structural factors that may discriminate against older adults in need of care, such as unaddressed needs or difficult-to-access healthcare services [32,86,88]. At the same time, many nurses in our sample developed a strong sense of responsibility for preventive care through deliberate moral reasoning. Thus, in line with Eriksson et al. [89], who describe how nurses in LTC settings address suicide prevention on multiple levels, as well as with Schwartz et al. [55] and Turiaux et al. [54], who report active involvement of German palliative care professionals in suicide prevention, our findings indicate that many nurses perceive suicide prevention as an integral aspect of their professional role.

Our findings underscore the ethical challenges nurses face when navigating requests for assisted suicide alongside responsibilities in suicide prevention. These challenges include reconciling diverse ethical perspectives or negotiating value-based conflicts between various stakeholders (moral controversy), coping with internal moral uncertainty when nurses are unsure about what they themselves consider the ethically right course of action, and managing (perceived) moral constraints (Theme 3. Ethical Challenges). Consistent with prior research [20,26,27,29,53,80], we observed a broad spectrum of ethical viewpoints on assisted suicide in our sample, ranging from full rejection to full support. This highlights the ongoing moral controversy regarding assisted suicide, for example, by showing that care recipients’ individual perspectives do not always align with other stakeholders’ ethical viewpoints, which may in turn reflect (perceived) broader societal moral norms (e.g., “*Self-determination* vs. *societal value norms, which may not align with individual beliefs*”; F5). In contrast, ethical perspectives on suicide prevention were less divergent, likely reflecting its lower moral controversy and greater societal acceptance. Divergent perspectives on MAiD, however, can create barriers to care, negatively affecting care recipients—for example, when healthcare professionals or entire institutions object to MAiD provision [45,75]—and may generate moral conflicts and tensions within teams, while also negatively impacting nurse–care recipient relationships [20,53]. Thus, one challenge lies in team communication, which previous studies have identified as a crucial resource for ethically managing MAiD [20,27,29]. Furthermore, good nurse-care recipient communication is particularly important, as relationship-building is central to suicide prevention [47], and meaningful conversations between nurses and care recipients may help alleviate suffering and potentially dissuade patients from pursuing MAiD [26]. Nevertheless, it must be acknowledged that care recipients may rightfully choose to undergo MAiD, despite efforts to alleviate their suffering—a choice that must be respected by nurses. We also identified value-based conflicts, particularly the moral dilemma of responding professionally to assisted suicide requests while simultaneously upholding responsibilities for suicide prevention. Similar dilemmas—balancing patient safety, understood as protection from risk and harm, against other ethical principles such as respect for autonomy or confidentiality—have been reported in prior research [47,48]. Nurses also reported how they experience a feeling of internal moral uncertainty, especially regarding assisted suicide, and to a lesser extent suicide prevention, which may indicate that they are continuously engaged in an ongoing process of moral sense-making (e.g., “*Shortening a person’s life—is it killing? Or helping? How do I deal with it myself?!!!*”; F6). This aligns with international findings and may be partly attributed to the lack of clear legal and professional guidance and unclear nursing roles and responsibilities in relation to MAiD [20,26,27,29,53]. Consistent with other research [20,26,27,47,56], participants expressed a need for further education to reduce uncertainty in both assisted suicide and suicide prevention. In addition, our findings highlight several external moral constraints. Some nurses perceived assisted suicide as legally impermissible in Germany, reflecting legal knowledge gaps also identified in other German [55] and international [27] studies underscoring the need for education. Particularly concerning, some nurses reported feeling obliged to participate in MAiD against their will, emphasizing the importance of conscientious objection, defined as “a healthcare professional’s refusal to participate in a legally authorized procedure due to deeply held personal beliefs” [90] (p. 8). Although conscientious objection remains a topic of ethical debate [90], this right is affirmed both legally and professionally as both the German Federal Constitutional Court [14] and the International Council of Nurses [43] explicitly point to this right. At the same time, it must be acknowledged that many healthcare professionals morally support MAiD, which is also a legal right in many countries. Thus, Peisah et al. [75] emphasize that, regardless of individual moral positions on MAiD, no one should be “named and shamed” (p. 1) for their moral stance. Instead, institutions must find ways to integrate MAiD practices that address the needs of caregivers holding various viewpoints on MAiD and care recipients alike. Regarding suicide prevention, participants also reported external barriers constraining morally appropriate practice, particularly insufficient time and limited access to specialized care. Resource constraints—especially time pressures [89]—have been identified as major challenges in suicide prevention. Time constraints are especially pressing given rising administrative demands and staffing shortages in LTC settings, which are currently recognized as critical challenges in Germany [91]. Limited access to healthcare services and resources—such as mental health care or palliative support —was identified by some nurses in our own and other studies as a concern in the context of MAiD [27,80,81,92]. Participants emphasized that better and more equitable access to these services could, in certain situations, help prevent MAiD. This highlights the need for societal changes to promote equal access to essential healthcare resources.

Overall, our findings—in the context of previous research and current political, societal and professional developments—suggest that nurses may experience moral distress when navigating the ethical challenges posed by assisted suicide requests and suicide prevention in care recipients who consider this option. Moral distress arises when nurses face psychological strain due to moral conflicts, tensions, dilemmas, uncertainty, or constraints [77,78]. Since all of these potential triggers as well as psychological stress were evident in our study, and given that moral distress related to MAiD has been documented internationally among nurses, it is likely that nurses in Germany and other countries encounter similar challenges [20]. This issue is particularly concerning because moral distress can have serious consequences for nurses—including physical and mental health impairments, job dissatisfaction, and staff turnover—as well as for care recipients, such as a reduction in the quality of care [93,94]. Accordingly, targeted measures are needed to support nurses in their moral role—both in responding professionally to MAiD requests and in suicide prevention. One approach to reducing moral distress is to strengthen nurses’ moral resilience, including their moral self-efficacy [49,52,95]. Importantly, strategies and interventions—such as educational programs—should be implemented across multiple levels: societal, professional, institutional, and team-based, rather than placing the responsibility solely on individual nurses [20,52,95]. Furthermore, since ethical perspectives on and attitudes towards assisted suicide and suicide prevention vary among nurses, interventions must also be tailored to individual needs [53] and take into account the range of moral stances on MAiD within healthcare teams [75]. Such measures could help safeguard both nurses and care recipients from the negative consequences of moral distress [20,49,52,95], promoting nurses’ well-being and enhancing the quality of care in the professional management of MAiD requests and suicide prevention.

### 4.2. Implications for Nursing Practice, Institutions, and Society

Based on our study findings and previous research, several practice implications emerge that should inform healthcare in Germany and are equally relevant for any country where MAiD is legal or under consideration.

At the individual level, nurses need to actively engage in strategies that foster the development of moral self-efficacy when addressing MAiD requests and suicide prevention. On the one hand, they should remain attentive to their personal moral stances including boundaries, seek available ethical support to guide their professional practice, and—if they hold a conscientious objection—communicate this accordingly in order to safeguard their moral integrity. On the other hand, it is important to ensure that individuals who choose MAiD continue to receive high-quality care, and that nurses recognize MAiD as a legally permissible option in many countries, which may reflect a person’s well-considered and autonomous decision. To effectively fulfill their moral responsibility in MAiD and suicide prevention care, nurses require support at the professional, institutional, and societal levels:

Firstly, at the professional and institutional level, there is a need for enhanced education and training. Educational programs should cover ethics training to foster moral resilience and moral self-efficacy, guidance on the right to conscientious objection and support for nurses’ moral responsibility to ensure that all care recipients receive high-quality nursing care—regardless of whether they choose MAiD. Programs should also address the legal and professional aspects of MAiD and suicide prevention care, as well as strategies to reduce ageism in healthcare [20,26,27,47,53,80,85]. Methods such as simulation training [86,96,97], drama-based workshops [98], debriefing, online resources [97] or the use of the evidence-based reflective guide introduced by Pesut et al. [99] may be effective for addressing these topics and should be integrated into nursing curricula as well as offered to practicing nurses as part of ongoing professional development.

Secondly, institutions should cultivate an ethical culture that supports personal and interprofessional team reflection on ethically challenging situations. Creating an environment that encourages open discussion of differing perspectives within healthcare teams could help reduce moral conflict and tension related to MAiD and suicide prevention [20,25,32,75]. As an example, Peisah et al. [75] introduce a useful strategy to deal with various moral stances on MAiD within healthcare teams and institutions. Where available, clinical ethicists can support healthcare professionals in their ethical decision-making through consultative or dialogical approaches (e.g., moral case deliberation) [100,101]. In home-based settings, where an ethicist or ethics committee is not always available, external ethics consultation services can offer a valuable alternative [91]. Alternatively, Frolic and Holland [102] present an ethics framework specifically adapted for MAiD-related ethical issues, which guides healthcare professionals through ethical reflection in a six-step process—even when professional ethicists are not accessible.

Thirdly, at both professional and institutional levels, nurses’ roles and responsibilities in MAiD and suicide prevention need clarification. Developing policies and ethical guidelines that address these areas, including conscientious objection, could provide a moral framework to guide nursing practice [20,26,47,53,80]. As an example, Isaac et al. [103] have developed a promising nine-step framework for conscientious objection that outlines the responsibilities of both employees and employers in balancing conscientious objections while ensuring continuity of care and protecting patient rights. Moreover, Bellon et al. [28] emphasize that nurses assume a wide range of roles and tasks throughout the MAiD process across different countries, which should be taken into account in guideline development. At the same time, policy and guideline frameworks need to emphasize interprofessional collaboration, as both MAiD and suicide prevention require coordinated, multidisciplinary practice [32,104,105]. The roles and responsibilities of various healthcare professionals (e.g., nurses, physicians, social workers) and services (e.g., palliative care, mental health) should be explicitly defined in such documents [106] to enhance integration among all stakeholders involved in MAiD and suicide prevention.

Fourthly, at the societal level, barriers to accessing health services that support suicide prevention should be addressed as a pressing public health issue [92], alongside efforts to strengthen the nursing workforce and mitigate reported time constraints [91]. This may be achieved through the development and implementation of national suicide prevention strategies in line with recommendations on goals and objectives by the World Health Organization [19] or through legal measures, such as the planned suicide prevention legislation in Germany [16], which, among other objectives, aims to enhance access to low-threshold support services. National nurses associations should—in line with ICN recommendations—“Lobby for working environments that promote healthy lifestyle standards for nurses” [43] (p. 13). At the same time, practicing nurses should actively advocate for ethical working conditions [91] and contribute their expertise on MAiD-related ethical issues and suicide prevention in older adults to interprofessional and policy discussions [29].

### 4.3. Strenghts and Limitations

A major strength of this study lies in its inclusion of a wide range of perspectives from nurses working in organizations of varying size and structure, representing both residential and home-based LTC. Furthermore, the use of a structured and theoretically underpinned coding framework supported the rigorous analysis of the data.

Yet, several limitations should be taken into account when interpreting our findings.

Although we captured a broad spectrum of ethical perspectives from nurses across different LTC organizations in the South and Mid-West of Germany, the transferability of our results to other German regions and other countries, legal frameworks and settings is limited. This is particularly true in view of the varying approaches to staffing, the diverse nurse qualification levels across countries, and the differing institutional framework conditions, ethical cultures and ethical climates [79]. Moreover, because we did not systematically collect sociodemographic data in order to safeguard participant anonymity, the transferability of our findings is further limited, as it becomes difficult to compare our sample with others.

Furthermore, while our aim was to explore a wide range of perspectives and challenges related to assisted suicide and suicide prevention, previous research has shown that participants may be hesitant to openly address such sensitive topics in group discussions [60]. Although we sought to reduce social desirability bias by conducting the focus groups in quiet, confidential rooms, emphasizing anonymity, affirming that there were no right or wrong opinions, providing context that MAiD and suicide prevention are professionally and ethically challenging for many healthcare professionals, and asking participants in each phase to first write down their responses individually before sharing them [82], it remains possible that they primarily discussed experiences they perceived as socially acceptable—particularly given that assisted suicide continues to be a highly controversial topic. In focus groups that included both frontline nurses, and nurses with additional leadership roles, hierarchical positions may have influenced participants’ responses.

Our method of data collection with no audio or video recordings must also be acknowledged, as the resulting data are inherently less concrete than recorded focus group discussions, with discursive nuances being lost. Although we sought to enhance interpretive accuracy by taking detailed field notes to capture the content, these notes may reflect the researcher’s own perceptions. While all researchers who participated in focus groups either as moderators or observers aimed to maintain a neutral stance and actively engaged in reflective practice regarding their own feelings, values, and attitudes (see postscripts), our findings may still be subject to annotation bias. Furthermore, internal validity may be limited because we did not provide participants with the full transcripts—including both their notes and the researcher’s field notes—for verification and therefore could not confirm whether the researcher’s interpretations accurately reflected participants’ intended meaning.

Additionally, technical challenges arose in the online focus groups as some participants experienced connectivity issues. As a result, certain potentially valuable insights may have been missed.

Finally, not all participants had direct experience with assisted suicide requests. Many instead drew on encounters with “desire to die” situations in reflecting on their practice, meaning that some responses regarding assisted suicide were necessarily hypothetical and should be interpreted accordingly.

### 4.4. Research Implications

Future research could adopt alternative research methods, such as audiorecorded individual interviews, to triangulate our results and to gain deeper and more nuanced insights into nurses’ ethical experience in the context of assisted suicide and suicide prevention in Germany. While our study primarily focused on nurses’ ethical perspectives and challenges, future research should explore strategies and practical approaches that nurses use to *navigate* ethically challenging situations in the context of MAiD and suicide prevention across countries. More research is needed to develop comprehensive strategies that enhance the integration of LTC services with medical and mental health care, focusing on interprofessional teamwork and integrated care models. Additionally, intervention studies are needed to examine the effects of measures such as educational programs designed to enhance nurses’ moral resilience and moral self-efficacy. Furthermore, it would be valuable to examine the underlying meanings and triggers of nurses’ emotional responses in relation to MAiD and suicide prevention in more detail. Finally, our findings suggest that suicide prevention can pose ethical challenges, particularly when considered alongside MAiD, yet research in this area remains limited. Thus, there is a need to investigate nurses’ ethical perspectives on suicide prevention for individuals who choose MAiD in greater depth, using qualitative approaches.

## 5. Conclusions

Requests for assisted suicide and suicide prevention present ethically complex situations for nurses in residential and home-based LTC in Germany. Nurses must navigate intuitive and emotional responses, analyze ethical perceptions, engage in ethical reflection, and confront numerous ethical challenges in these contexts.

This study adds to existing knowledge by providing a deeper understanding of how such situations may contribute to the development of moral distress, negatively impacting nurses’ well-being and potentially compromising the quality of patient care in Germany and other countries where MAiD is legal. The ethical challenges and moral values identified may inform the development of ethical guidelines specifically tailored to the practical challenges faced by German LTC nurses in providing care related to assisted suicide and suicide prevention. The study contributes to the international debate by highlighting the ethical challenges nurses face in providing professional MAiD care, respecting patients’ autonomous decisions and legal rights, while simultaneously bearing responsibility for suicide prevention.

Educational interventions, the cultivation of an open ethical climate, clear role definitions, guidelines and the removal of barriers to suicide prevention may help reduce nurses’ moral distress, both in Germany and internationally. Taken together, these measures can safeguard moral integrity, strengthen moral resilience, including moral self-efficacy, and provide a more stable ethical framework. This, in turn, enables nurses to respond professionally to MAiD requests while effectively supporting suicide prevention, ultimately benefiting both nurses’ well-being and the quality of patient care.

## Figures and Tables

**Table 1:** Focus Groups by Setting, Format, and Participant Numbers.

	Residential LTC		Home-Based LTC	
	Number of Focus Groups	Number of Participants	Number of Focus Groups	Number of Participants
In-Person Focus Group	*n* = 7	*n* = 62	*n* = 3	*n* = 17
Online Focus Group	*n* = 1	*n* = 11	*n* = 1	*n* = 6
Total	*n* = 8	*n* = 73	*n* = 4	*n* = 23

## Data Availability

The data presented in this study are available from the corresponding author upon reasonable request, as they form part of an ongoing research project.

## Supplementary Materials

The following supporting information can be downloaded at: https://www.mdpi.com/article/10.3390/healthcare13243263/s1, Supplementary File S1: COnsolidated criteria for REporting Qualitative studies (COREQ): 32-item Checklist; Supplementary File S2: Focus Group Interview Guide.

## Abbreviations

The following abbreviations are used in this manuscript:

LTC	Long-Term Care
MAiD	Medical Assistance in Dying
