# Supplementary material for: Assisted Suicide and Suicide Prevention: Ethical Perspectives, Attitudes and Challenges for Nurses in Long-Term Care—A Qualitative Focus Group Study
Source: Healthcare (Basel). 2025 Dec 12;13(24):3263. doi: 10.3390/healthcare13243263 (PMC12732559; doi:10.3390/healthcare13243263)
Supplement: Supplementary file 1 [file healthcare-13-03263-s001.zip › Supplementary File 2_Healthcare_Klotz et al.pdf]

Supplementary File 2: Focus Group Interview Guide

Assisted Suicide and Suicide Prevention: Ethical Perspectives, Attitudes and Challenges for Nurses in Long-Term Care— A Qualitative Focus Group Study

Karen Klotz *, Pia Madeleine Haug, Thomas Heidenreich, Eva-Maria Stratmann, Erik Jacob and Annette Riedel

Faculty of Social Work, Education and Nursing Sciences, Esslingen University of Applied Sciences, Flandernstraße 101, 73732 Esslingen, Germany; pia-madeleine.haug@hs-esslingen.de (P.M.H.); thomas.heidenreich@hs-esslingen.de (T.H.); eva-maria.stratmann@hs-esslingen.de (E.-M.S.); erik.jacob@hs-esslingen.de (E.J.); annette.riedel@hs-esslingen.de (A.R.)

* Correspondence: karen.klotz@hs-esslingen.de

**Content & Topics**	**Duration (Total: 120–150 min)**	**Questions & Assignments**	**Methods & Materials**	**Aims & Objectives**
**Phase 1: Welcome and Settling In**				
Welcome and thanks<br><br>Mutual introductions (researchers and participants)<br><br>Information about the research project, the procedure, the goal, and the duration of the focus group<br><br>Information on voluntariness throughout the entire process<br><br>Information on anonymity in the context of data collection, documentation, analysis and result reporting<br><br>Request for confidentiality<br><br>Information on support opportunities in dealing with distressing material. In case of a potential crisis elicited by exposure to stressful material during focus group, crisis intervention will be provided by TH (licensed psychotherapist)<br><br>Brief explanation of the focus group method and the focus of the group discussion	10 min	Is anything still unclear or unresolved?<br><br>Do you have any remaining uncertainties or doubts?<br><br>Are you able to be fully present?	Participants script:<br>→ Written communication rules for discussion (e.g., do not mention names of real persons or institutions, stay focused on your own experience, do not interrupt,…)	A confidential atmosphere is created, where an initial getting-to-know-each-other can take place upon arrival (coffee, pastries)<br><br>Participants are thoroughly informed about the research process and the data collection method<br><br>The procedure is transparent<br><br>Participants are informed about their rights, including data protection, anonymity, confidentiality, and the voluntary nature of participation<br><br>Participants are informed about available support options in case a situation is experienced as psychologically distressing<br><br>Participants are introduced to the data collection method<br><br>Written and verbal informed consent to participate is obtained

Content & Topics	Duration (Total: 120–150 min)	Questions & Assignments	Methods & Materials	Aims & Objectives
Communication rules for discussion<br><br>Informed written and verbal consent				
**Phase 2: Orienting Participants to the Focus and Topic of the Focus Group**				
Definition of assisted suicide (here in distinction from euthanasia; in the context of the overarching phenomenon of desire to die; legal context in Germany)<br><br>Definition of suicide prevention	5–10 min	Are there any remaining questions?<br><br>Is there anything you want to clarify?	Participants script:<br>→ Written definition of assisted suicide<br>→ Written definition of suicide prevention	Participants are familiar with the basic terminology on phenomena discussed in the focus group<br><br>The focus of the group discussion is clarified (assisted suicide and suicide prevention)
**Phase 3: Establishing the Link to Practice/Personal Experience**				
Case examples (drawn from personal experience/professional nursing practice)	5–10 min	Can you recall a situation from your nursing practice in which the topic of a request for assisted suicide played a role?<br><br>If not: Can you recall a situation in which a desire to die played a role?<br><br>If not: Prepared case example<br><br>→ Make some notes on a facilitation card. Keep them with you for now.	Participants script<br><br>Case example (Backup)<br><br>Facilitation cards	Introduction to lived professional experience

Content & Topics	Duration (Total: 120–150 min)	Questions & Assignments	Methods & Materials	Aims & Objectives
**Phase 4: Contextualizing Moral Self-Efficacy**				
Definition of moral self-efficacy<br><br>Participants complete the online questionnaire on moral self-efficacy to support reflection<br><br>Important: Participation in the online questionnaire is voluntary! Online questionnaire can be used without submitting it for data processing<br><br>Once participants have finished, they can place a sticky dot on initially hidden sheets<br><br>Participants may still place a sticky dot even if they do not complete or submit the online questionnaire	15 min	Based on your reflection: How would you currently rate your own moral self-efficacy regarding requests for assisted suicide?<br><br>→ Place a sticky dot on prepared sheets (Arrow ranging from “low moral self-efficacy” <---------> “high moral self-efficacy” in relation to dealing with assisted suicide requests/suicide prevention in individuals who request assisted suicide<br><br>(At the end of the focus group, the sheets will be reviewed to identify next steps in the research project, which seeks to enhance moral self-efficacy in handling assisted suicide requests and suicide prevention)	Participants script:<br>→ Definition of moral self-efficacy<br>→ Link for online questionnaire on moral self-efficacy<br>→ Prepared sheets (moral self-efficacy)	Transition to the significance of the ethical perspective and moral discomfort – moving beyond purely technical or professional uncertainties<br><br>Identification and relief at the professional/technical level<br><br>Capturing and reflecting upon moral self-efficacy as a professional phenomenon that may help in dealing with ethically challenging situations<br><br>Prerequisite and transition to the next step
**Phase 5: Building on Practice-Oriented Associations**				
Participants’ experiences and perceptions regarding requests for assisted suicide and suicide prevention	15 min	Thinking about your own/the case example: What do you associate with these or similar situations (emotions and thoughts)?<br>a. When thinking about requests for assisted suicide?<br>b. When thinking about suicide prevention?	Participant script<br><br>Facilitation cards	Participants begin to engage with their own experiences and personal perceptions<br><br>Initial associations and resonances have been captured

Content & Topics	Duration (Total: 120–150 min)	Questions & Assignments	Methods & Materials	Aims & Objectives
		→ Write down these emotions and thoughts on the facilitation cards. Keep them with you for now.<br>What situations and associations (Phase 3 & 5) came up for you?<br><br>→ Present situations and associations to group and discuss		
**Phase 6: Specification of Professional and Ethical Challenges**				
The professional and ethical challenges are identified and articulated<br><br>Participants place and present the facilitation cards with the challenges they have formulated in the plenary session, sharing those they wish to discuss	20 min	Which challenges do you consider relevant for discussion?<br>a. When thinking about requests for assisted suicide?<br>b. When thinking about suicide prevention?<br>→ Note down key points on 2–4 facilitation cards for each<br><br>Which of the noted challenges can be categorized as professionally relevant for discussion?<br>Which of the noted challenges can be categorized as ethically relevant for discussion?<br>Where is a clear categorization possible?<br>Where is no clear categorization possible?<br>→ Place your facilitation cards in the appropriate location on the poster and discuss	Participant script<br><br>Poster (Professional vs. Ethical Challenges vs. Professional and Ethical Challenges)	Clarifying the complexity<br><br>Capture ethical and professional uncertainties, knowledge gaps, challenges, and similar issues<br><br>Clarifying the distinction between the professional and the ethical perspective

Content & Topics	Duration (Total: 120–150 min)	Questions & Assignments	Methods & Materials	Aims & Objectives
**Phase 7: Elaborating on the Ethical Dimension**				
Theoretical input: What are values in nursing?<br><br>Relevant values are identified<br><br>Ethical value conflicts are explored in small groups	35 min	Individual work (Step 1):<br>Which values are involved and affected in the context of requests for assisted suicide/suicide prevention? (Feel free to think about your case example; you may also refer back to your thoughts and emotions from Phase 5.)<br><br>Which values would you like to note in your individual square?<br><br>→ Differentiate which person from the case example each value is associated with.<br>Which of these values might be in conflict with one another?<br><br>Small group discussion (3–4 participants each; Step 2):<br>→ Share your results with each other, then decide which value conflict represents the central ethically relevant conflict for your small group (place it in the center of the poster)<br><br>Plenary presentation and discussion:<br>→ Each small group presents their results	Participant Script<br>→ Definition and catalogue of values<br><br>Placemat-method: prepared poster (group work)	The values involved are systematically identified and differentiated<br><br>A basis is established for identifying value conflicts<br><br>Value conflicts have been captured<br><br>The central ethically relevant value conflicts for the small groups and entire focus group have been identified

**Content & Topics**	**Duration (Total: 120–150 min)**	**Questions & Assignments**	**Methods & Materials**	**Aims & Objectives**
		Based on the formulations, which value conflict should be considered the central conflict for the entire group?<br>→ Discuss (entire focus group)		
**Phase 8: Closing and Outlook**				
Outlook – Support methods developed within the research project (practice and ethics guidelines). Look back at moral self-efficacy sheets – point out, that one aim of the research project is to improve moral self-efficacy when dealing with assisted suicide requests/suicide prevention<br><br>Collecting hopes and expectations regarding the guidelines to be developed in the research project<br><br>Note to participants: At the end, there is another opportunity to withdraw facilitation cards that you do not wish to be included in the data analysis<br><br>Support offers: reference to ethical case discussions and psychosocial/emotional support options<br><br>Farewell and Thanks	10 min	What do you hope to gain from the practice and ethics guidelines to be developed within the research project?<br>→ Please place a sticky dot in the appropriate location<br><br>Anything else?<br>→ Write any additional hopes or expectations not listed on a facilitation card<br><br>How are you leaving the focus group?<br><br>Are there facilitation cards on the boards/posters that you do not want included in the data analysis?<br>→ You have the option to withdraw facilitation cards. These will then not be included in the analysis	Participant script<br><br>Moral self-efficacy sheets (anonymized)<br><br>Sticky dots<br><br>Facilitation cards	Transparency regarding data usage<br><br>Ensuring that everyone feels supported and aware of available (moral) relief formats<br><br>Re-confirming consent to data processing (verbally)
