# Supplementary material for: Assisted Suicide and Suicide Prevention: Ethical Perspectives, Attitudes and Challenges for Nurses in Long-Term Care—A Qualitative Focus Group Study
Source: Healthcare (Basel). 2025 Dec 12;13(24):3263. doi: 10.3390/healthcare13243263 (PMC12732559; doi:10.3390/healthcare13243263)
Supplement: Supplementary file 1 [file healthcare-13-03263-s001.zip › Supplementary File 1_Healthcare_Klotz et al_Rev.pdf]

Supplementary File 1 – COnsolidated criteria for REporting Qualitative studies (COREQ): 32-item Checklist

Assisted Suicide and Suicide Prevention: Ethical Perspectives, Attitudes and Challenges for Nurses in Long-Term Care—
A Qualitative Focus Group Study

Karen Klotz *, Pia Madeleine Haug, Thomas Heidenreich, Eva-Maria Stratmann, Erik Jacob and Annette Riedel

Faculty of Social Work, Education and Nursing Sciences, Esslingen University of Applied Sciences,
Flandernstraße 101, 73732 Esslingen, Germany; pia-madeleine.haug@hs-esslingen.de (P.M.H.);
thomas.heidenreich@hs-esslingen.de (T.H.); eva-maria.stratmann@hs-esslingen.de (E.-M.S.);
erik.jacob@hs-esslingen.de (E.J.); annette.riedel@hs-esslingen.de (A.R.)
* Correspondence: karen.klotz@hs-esslingen.de

This table was derived from the COnsolidated criteria for REporting Qualitative studies (COREQ) checklist by Tong et al. [62][1].

No	Item	Question/Description	Page
**Domain 1: Research Team and Reflexivity**			
**Personal Characteristics**			
1	Interviewer/Facilitator	*Which author/s conducted the interview or focus group?*	p. 5
2	Credentials	*What were the researcher's credentials? E.g. PhD, MD*	p. 7
3	Occupation	*What was their occupation at the time of the study?*	p. 7
4	Gender	*Was the researcher male or female?*	p. 5
5	Experience and Training	*What experience or training did the researcher have?*	p. 7
**Relationships with Participants**			
6	Relationship Established	*Was a relationship established prior to study commencement?*	p. 7
7	Participant Knowledge of the Interviewer	*What did the participants know about the researcher? e.g. personal goals, reasons for doing the research*	p. 7
8	Interviewer Characteristics	*What characteristics were reported about the interviewer/facilitator? e.g. Bias, assumptions, reasons and interests in the research topic*	p. 7
**Domain 2: Study Design**			
**Theoretical Framework**			
9	Methodological Orientation and Theory	*What methodological orientation was stated to underpin the study? e.g. grounded theory, discourse analysis, ethnography, phenomenology, content analysis*	p. 3
**Participant Selection**			
10	Sampling	*How were participants selected? e.g. purposive, convenience, consecutive, snowball*	p. 4
11	Method of Approach	*How were participants approached? e.g. face-to-face, telephone, mail, email*	p. 4
12	Sample Size	*How many participants were in the study?*	p. 4–8
13	Non-Participation	*How many people refused to participate or dropped out? Reasons?*	p. 4
14	Setting of Data Collection	*Where was the data collected? e.g. home, clinic, workplace*	p. 4
15	Presence of Non-Participants	*Was anyone else present besides the participants and researchers?*	p. 5
**16**	Description of Sample	*What are the important characteristics of the sample? e.g. demographic data, date Data collection*	p. 4-8
**Data Collection**			
17	Interview Guide	*Were questions, prompts, guides provided by the authors? Was it pilot tested?*	p. 4–5, Supplementary File 2

[1] For reference details, please consult the main manuscript.

18	Repeat Interviews	*Were repeat interviews carried out? If yes, how many?*	p. 4
19	Audio/Visual Recording	*Did the research use audio or visual recording to collect the data?*	p. 5
20	Field Notes	*Were field notes made during and/or after the interview or focus group?*	p. 5
21	Duration	*What was the duration of the interviews or focus group?*	p. 4
22	Data Saturation	*Was data saturation discussed?*	p. 5–6
23	Transcripts Returned	*Were transcripts returned to participants for comment and/or correction?*	p. 5
**Domain 3: Analysis and Findings**			
**Data Analysis**			
24	Number of Data Coders	*How many data coders coded the data?*	p. 6
25	Description of the Coding Tree	*Did authors provide a description of the coding tree?*	p. 8–10
26	Derivation of Themes	*Were themes identified in advance or derived from the data?*	p. 6–7
27	Software	*What software, if applicable, was used to manage the data?*	p. 6
28	Participant checking	*Did participants provide feedback on the findings?*	p. 5
**Reporting**			
29	Quotations Presented	*Were participant quotations presented to illustrate the themes / findings? Was each quotation identified? e.g. participant number*	p. 8–14
30	Data and Findings Consistent	*Was there consistency between the data presented and the findings?*	p. 8–14
31	Clarity of major themes	*Were major themes clearly presented in the findings?*	p. 8–14
32	Clarity of minor themes	*Is there a description of diverse cases or discussion of minor themes*	p. 8–14
